# Screening of Differentially Expressed Genes Related to Growth, Development and Meat Quality Traits of Huanghuai Sheep Based on RNA-Seq Technology

**DOI:** 10.3390/ani15243612

**Published:** 2025-12-15

**Authors:** Wanli Han, Mengke Song, Fuxian Gao, Haoyuan Han, Huibin Shi, Kai Quan, Jun Li

**Affiliations:** 1 College of Animal Science and Technology, Henan University of Animal Husbandry and Economy, Zhengzhou 450046, China; ahan401@163.com (W.H.); songmengke1113@163.com (M.S.); gaofx0506@163.com (F.G.); hanhaoyuan@126.com (H.H.); huibinshi0715@163.com (H.S.); quankai1115@163.com (K.Q.); 2 Faculty of Animal Science and Technology, Yunnan Agricultural University, Kunming 650201, China

**Keywords:** Huanghuai sheep, growth and development, meat quality traits, *Longissimus dorsi*, transcriptome, correlation analysis

## Abstract

Huanghuai sheep, a newly developed meat-specialized breed in China, are valued for their rapid growth and high meat quality, but the optimal slaughter age and the molecular basis of these traits remain poorly understood. Gaining insight into these mechanisms is vital for improving production efficiency and guiding molecular breeding in this economically important breed. Although previous studies have described the phenotypic characteristics of Huanghuai sheep, the genetic regulatory networks controlling muscle growth and meat quality at different developmental stages remain unclear. No thorough analysis of growth traits and transcriptomic variations across key age points has been conducted. Therefore, in this study, we aimed to evaluate how growth stage influences muscle development, carcass characteristics, and meat quality in Huanghuai sheep by integrating phenotypic characterization with transcriptomic profiling to identify key genes and molecular pathways underlying these economically important traits throughout development. Sixty Huanghuai sheep were assigned to three groups (twenty per group) representing key developmental stages (3, 9, and 18 months of age). Carcass traits and meat quality were evaluated. RNA sequencing of the *longissimus dorsi* muscle was performed to identify differentially expressed genes (DEGs), followed by bioinformatics analysis and experimental validation. The results indicated that the 9-month-old sheep presented a favorable balance of dressing percentage and intramuscular unsaturated fatty acid content, while those aged 18 months old exhibited the highest dressing percentage (61.23%). Transcriptome analysis identified 1395 DEGs (*p* < 0.05 and |log2FC| > 1) and enrichment analysis revealed key pathways involved in thyroid hormone synthesis, skeletal muscle satellite cell proliferation, and skeletal muscle tissue growth. Several candidate genes for muscle development (e.g., *ACTC1*, *SIX2*, *HK2*) and meat quality (e.g., *TLR2*, *CHI3L1*, *ACOT7*) were identified and validated. Their expression patterns showed significant correlations between critical growth performance and fatty acid composition metrics. These findings provide novel insights into the molecular networks regulating economically important traits in Huanghuai sheep, offering valuable targets for future molecular breeding programs aimed at enhancing productivity and meat quality.

## Simple Summary

The Huanghuai sheep are a newly developed Chinese breed characterized by high fertility, rapid growth rate, and superior meat quality. In this study, we investigated how muscle development and meat quality change at different ages (3, 9, and 18 months). The results showed that 9-month-old sheep had the best meat quality, while those aged 18 months old had the highest meat yield. RNA sequencing identified key genes linked to muscle growth and meat quality. These findings elucidate the genetic basis of muscle development and meat quality in Huanghuai sheep, which provides valuable insights and a practical reference for optimizing slaughter timing and improving breeding strategies in the sheep industry.

## 1. Introduction

Lamb meat is valued as an important source of high-quality protein and essential nutrients [1]. In sheep breeding, enhancing meat yield and quality—such as body weight, dressing percentage, intramuscular fat (IMF) content, and fatty acid profiles—remains a primary goal, as meat production performance is a critical determinant of livestock productivity and a paramount economic metric guiding genetic improvement programs [2,3]. However, the genetic complexity of these polygenic traits limits the efficiency of conventional breeding methods.

The Huanghuai sheep are a newly developed specialized meat breed that represents a significant achievement in China’s domestic livestock breeding programs [4]. Systematically developed through multi-generational selection, this breed has robustly adapted to the natural resources and climatic environment of the Huanghuai Plain. It is characterized by several primary advantages, including desirable carcass meat quality, high fecundity, and rapid growth and development rates, which constitute its critical economic value and have made it an important genetic resource for mutton production in the Huang-Huai region [5]. Although its phenotypic advantages are well-recognized, molecular-level insights into these traits have lagged behind. Existing studies have primarily focused on germplasm characterization and production performance, leaving the transcriptomic foundations of its superior meat production virtually unexplored. This gap significantly constrains targeted genetic improvement. While transcriptomic studies in other sheep breeds have highlighted several conserved pathways, such as ECM–receptor interaction [6,7], PI3K-Akt [8,9], and PPAR signaling [10,11], as crucial regulators of muscle development and lipid metabolism, the extent to which these mechanisms operate in Huanghuai sheep, and what breed-specific regulators may exist, are open questions.

Through this study, we have bridged this gap by integrating phenotypic data with transcriptome analysis of the *longissimus dorsi* across three key developmental stages (3, 9, and 18 months), which represent distinct physiological transitions: rapid myofiber growth, initiation of intramuscular fat deposition, and the commercial fattening phase, respectively. We hypothesized that stage-specific transcriptional dynamics underpin the shifts in growth performance, meat quality, and fatty acid composition observed across these stages. To test this, we aimed to (1) identify differentially expressed genes (DEGs) associated with developmental transitions and (2) evaluate the correlation between gene expression and key traits—including growth and slaughter metrics, meat quality, and fatty acid profiles—using Pearson’s correlation analysis. Our findings provide a molecular basis for the genetic improvement of Huanghuai sheep and support the development of precision breeding strategies in the sheep industry.

## 2. Materials and Methods

### 2.1. Ethics Statement

All procedures were approved by the Animal Welfare Committee of the College of Animal Science and Technology, Henan University of Animal Husbandry and Economy (HNUAHE ER 2425107, 25 June 2024), and were conducted in accordance with institutional and local animal care guidelines.

### 2.2. Collection of Animal Samples

A total of sixty purebred male Huanghuai sheep were randomly selected from the Huanghuai sheep original breeding farm (Henan Lvyuan Meat Sheep Development Co., Ltd., Luohe, China). The sheep were allocated into three age groups 3 months (*n* = 20), 9 months (*n* = 20), and 18 months (*n* = 20), with each group representing a distinct developmental stage.

All animals were managed uniformly under intensive housing conditions. They were fed a total mixed ration (TMR) consisting primarily of peanut vine as roughage, supplemented with concentrated feed (including corn, soybean meal, wheat bran, and premix) formulated to meet specific nutritional requirements at each developmental stage. Water was available ad libitum. A strict health assessment was conducted prior to selection, which included evaluation of body condition, alertness, appetite, fecal consistency, rectal temperature, and the absence of clinical signs of respiratory or systemic disease.

### 2.3. Growth and Development Traits and Meat Quality

Growth and development indicators (body height, body length, chest circumference, cannon circumference, and body weight) of each experimental sheep were measured in all twenty experimental sheep per age group. Following this, three Huanghuai sheep from the 3-month-old and 18-month-old stages as well as six from the 9-month-old stage were randomly selected for slaughter. After exsanguination via the jugular vein and removal of the head, hooves, skin, and viscera (with kidneys retained), the hot carcass weight was immediately recorded. Approximately 65 g of *longissimus dorsi* samples obtained from between the 12th and 13th ribs were cooked at 71 °C for 35 min in a thermostatically controlled water bath (HH-4, Weipinyiqi Precision Instruments Co., Ltd., Shenzhen, China) and then blotted dry for reweighing. The 12 samples were processed in three batches. After overnight cooling at 4 °C, shear force was measured with a crosshead speed of ≤ 5 mm/s using a tenderometer (C-LM4, Beijing Brabender Technology Development Co., Ltd., Beijing, China) on 1 cm × 1 cm × 1.5 cm cubes. Each sample was analyzed in triplicate. Meat production performance and quality traits, such as dressing percentage, marbling, pH, tenderness, and GR value, were then assessed according to the industry standard T/CAAA 080—2022 “Technical Specifications for Performance Testing of Meat Sheep”[12].

### 2.4. Determination of Fatty Acids

A 200 g sample of the *longissimus dorsi* was collected, and the IMF content was determined according to GB/T 41366—2022 “Determination of Moisture, Protein, and Fat Content in Livestock and Poultry Meat by Near-Infrared Spectroscopy”[13].The fatty acid composition was determined using gas chromatography–mass spectrometry (GC-MS). Fifty milligrams of the sample was weighed and derivatized with sulfuric acid–methanol for 30 min to convert fatty acids into fatty acid methyl esters (FAMEs). The FAMEs were then extracted with a mixture of chloroform–methanol (2:1, *v*/*v*) and n-hexane. After extraction, the solution was washed with water to remove impurities.

The gas chromatography analysis was performed on a system equipped with a flame ionization detector (FID) and an RBX-FAME capillary column (100 m × 0.25 mm × 0.25 μm). The injection volume was 1.0 μL in split mode with a split ratio of 20:1. The injector temperature was set at 270 °C. High-purity nitrogen was used as the carrier gas at a constant flow rate of 0.8 mL/min. The detector temperature was maintained at 270 °C. The detector gas flows were set as follows: hydrogen at 30 mL/min, nitrogen (make-up gas) at 30 mL/min, and air at 300 mL/min. The oven temperature was programmed as follows: initial temperature of 100 °C held for 13 min, increased to 180 °C at a rate of 5 °C/min and held for 8 min, then raised to 200 °C at 2 °C/min and held for 10 min, and finally increased to 230 °C at 10 °C/min and held for 10 min. The total run time was 68 min.

For quantification, undecanoic acid (C11:0) was used as the internal standard. The individual fatty acids were identified by comparing their retention times with those of known standards. The content of each fatty acid was calculated by comparing its peak area to that of the internal standard and was expressed as a percentage of the total fatty acids identified.

### 2.5. Total RNA Extraction

Total RNA was extracted from 100 mg of *longissimus dorsi* muscle of nine Huanghuai sheep (*n* = 3 for each group) using TRIzol reagent (TianGen, Beijing, China). The RNA quality and quantity were assessed using a NanoDrop 2000 Spectrophotometer and an Agilent 2100 Bioanalyzer (Agilent Technologies, Santa Clara, CA, USA). The RNA samples with an RNA Integrity Number (RIN) value > 7.0 were used for cDNA library construction. Genomic DNA contamination was eliminated, and first-strand cDNA synthesis was performed using a two-step reverse transcription protocol (Takara, Beijing, China). Sequencing libraries were prepared using the Hieff NGS^®^ Ultima Dual-mode mRNA Library Prep Kit for Illumina (Yeasen Biotechnology, Shanghai, China) according to the manufacturer’s protocol. Nine samples were sequenced using the Illumina NovaSeq X Plus platform (Illumina, San Diego, CA, USA) manufactured by Beijing Biomarker Biotechnology Co., Ltd. (Beijing, China).

### 2.6. RNA Sequencing

The raw data cleaning was filtered by fastp (version 21.0). The detailed process included removing linkers and removing reads that contained more than 10% N and low-quality reads (the number of bases with quality value Q ≤ 10 accounts for more than 50% of the entire read) to obtain high-quality clean reads. These clean reads were aligned to the sheep reference genome (Oar_v2.0) using HISAT 2 (version 2.0.4), generating alignment results per sample. The software Stringtie (version 2.2.1) was used to reconstruct the transcripts. All software used default parameters.

### 2.7. Data Processing

For quantitative and statistical analyses of gene expression levels for each sample group, the FPKM (Fragments Per Kilobase of transcript per Million mapped reads) algorithm was used to measure the expression levels of transcripts or genes. DEGs were identified based on the criteria of *p*-value < 0.05 and |log_2_ FC| > 1 [14]. The DESeq 2 pack in R (version 1.30.1) was used to generate volcano plots and expression heatmaps.

### 2.8. GO and KEGG Functional Enrichment Analysis

GO and KEGG functional enrichment analyses of DEGs were performed using the ClusterProfiler and pathview packages in R software (version 4.3.1), respectively. A significance threshold of *p* < 0.05 was applied to determine the statistical significance of enriched Gene Ontology (GO) terms and Kyoto Encyclopedia of Genes and Genomes (KEGG) pathways. Visualizations for the results of both GO and KEGG functional enrichment analyses were generated using the ggplot2 package in R. We focused on the GO terms that related to growth, development, and meat quality in the sheep.

### 2.9. Quantitative Real-Time PCR Validation

The reliability of mRNA sequencing results from different age groups of Huanghuai sheep was verified by qRT-PCR. Five DEGs (*ACTC1*, *ANKRD1*, *SIX2*, *GPC3*, and *HK2*) were randomly selected for validation, with glyceraldehyde-3-phosphate dehydrogenase (*GAPDH*) serving as the internal control [15]. *GAPDH* was used as the reference gene because its expression showed minimal variation across developmental stages in our RNA-seg data, and it is widely validated in livestock studies as a stable housekeeping gene unaffected by age-related physiological changes. The primers used in this study were designed by Primer 5.0 software (Table 1) and were synthesized by Sangon Biotech (Shanghai, China). qRT-PCR was performed on the Applied Biosystems QuantStudio 6 Pro Real-Time PCR System (Thermo Fisher Scientific, Waltham, MA, USA) using PowerUp™ SYBR™ Green Master Mix (Thermo Fisher Scientific, Waltham, MA, USA). RNA extracted, as described in Section 2.2 (1 μg), was converted to cDNA using FastKing gDNA Dispelling RT SuperMix (TinaGen, Beijing, China), and the cDNA was used as a template for qRT-PCR. The qRT-PCR reaction mixture was 20 μL, comprising 2 × SuperReal PreMix Plus 10 μL, 50 × ROX Reference Dye 0.4 μL, forward reverse primers 0.5 μL, cDNA 0.8 μL, and ddH_2_O 7.6 μL. The thermal cycling protocol included an initial pre-denaturation step at 95 °C for 15 min, followed by 40 cycles of denaturation at 95 °C for 10 s and extension at 60 °C for 30 s. The qRT-PCR results were calculated using the 2^−∆∆Ct^ method, and each sample was tested three times.

### 2.10. Statistical Analysis

The sample size for statistical analyses varied according to the trait measured: growth traits were evaluated using all 20 biological replicates per age group; slaughter and meat quality traits were assessed using the 12 slaughtered animals (3, 6, and 3 per age group, respectively); and transcriptomic analysis was performed with 3 biological replicates per group for RNA-seq. All statistical analyses were performed using SPSS 26.0 Statistics. Prior to analysis, the normality of the data distribution was verified using the Shapiro–Wilk test, and homogeneity of variances was confirmed using Levene’s test. The qPCR validation result was illustrated using GraphPad Prism 9.5. For comparisons across multiple age groups (3, 9, and 18 months), a one-way analysis of variance (ANOVA) was used, followed by Tukey’s post hoc test for multiple comparisons. The Pearson correlation coefficient was employed to assess the relationships between target gene expression levels and meat quality traits. Correlation *p*-values were adjusted for multiple testing using the Benjamini–Hochberg false discovery rate (FDR) procedure where applicable. Differences were considered statistically significant at p<0.05, and highly significant at p<0.01.

## 3. Results

### 3.1. Growth and Development Traits

As shown in Table 2, the carcass traits of Huanghuai sheep exhibited significant age-dependent variations across growth stages; morphological traits—including body height, body length, and chest circumference—showed highly significant intergroup differences (*p* < 0.01), while production performance indicators such as cannon circumference and body weight demonstrated significant differences (*p* < 0.05).

### 3.2. Meat Quality

As shown in Table 3, it was found that the meat quality of Huanghuai sheep exhibited distinct variations dependent on age across the growth stages: the carcass weight, dressing percentage, marbling, and GR value showed highly significant intergroup differences (*p* < 0.01). The GR value increased with age, following a significant increase between 9 and 18 months, which indicated pronounced fat deposition at 18 months of age.

### 3.3. Fatty Acid Content

As shown in Table 4, fatty acid content measurements in the *longissimus dorsi* showed that the levels of C14:0, C20:1, C20:2, C24:0, and C23:0 were significantly higher in 3-month-old sheep compared to those at 18 months (*p* < 0.05), while the C18:1n9c content was significantly lower in 3-month-old sheep compared to those at 9 and 18 months (*p* < 0.05). The content of C16:0, C16:1, C18:0, C18:2n6c, C18:3n3, and C20:4n6 in the muscles of Huanghuai sheep at 3 months, 9 months, and 18 months of age showed no significant differences between groups.

### 3.4. Transcriptome Sequencing Data Analysis

Using the *longissimus dorsi* tissues of Huanghuai sheep at 3, 9, and 18 months of age as samples, nine cDNA libraries were constructed. Following quality control of the raw sequencing data (Table 5), a total of 214,965,572 raw reads and 211,324,916 clean reads were obtained, with an average of 23,480,546 clean reads per sample and 63.26 Gb of effective bases. The GC content of each sample ranged from 50.14% to 52.34%, with Q30 ≥ 94.25%, indicating high base-calling accuracy. The high-quality sequences were aligned to the sheep reference genome (Oar_v2.0), with alignment rates ranging from 97.25% to 97.90% and an average of 92.22% of sequences being uniquely mapped. These results demonstrate the accuracy and reliability of the transcriptome sequencing data.

### 3.5. Analysis of Differentially Expressed Genes

**Figure 1:**
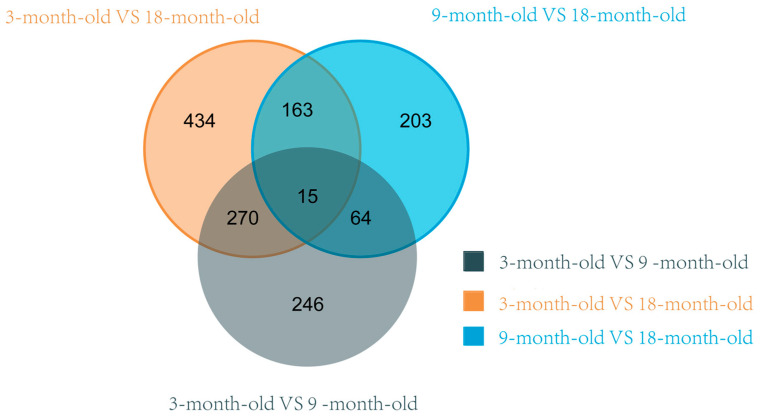
Venn diagram of co-expressed genes in longissimus dorsi tissue of Huanghuai sheep.

**Figure 2:**
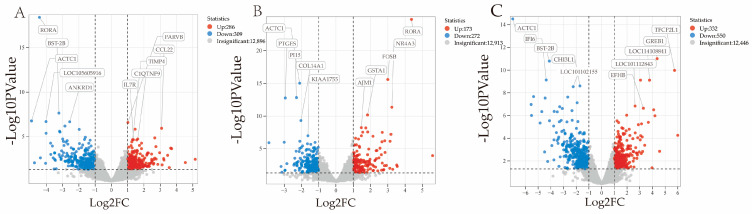
Analysis of gene expression in the longissimus dorsi muscle of Huanghuai sheep. (**A**) Volcano map of DEGs at the 3-month-old stage vs. the 9-month-old stage. (**B**) Volcano map of DEGs at the 9-month-old stage vs. the 18-month-old stage. (**C**) Volcano map of DEGs at the 3-month-old stage vs. the 18-month-old stage.

**Figure 3:**
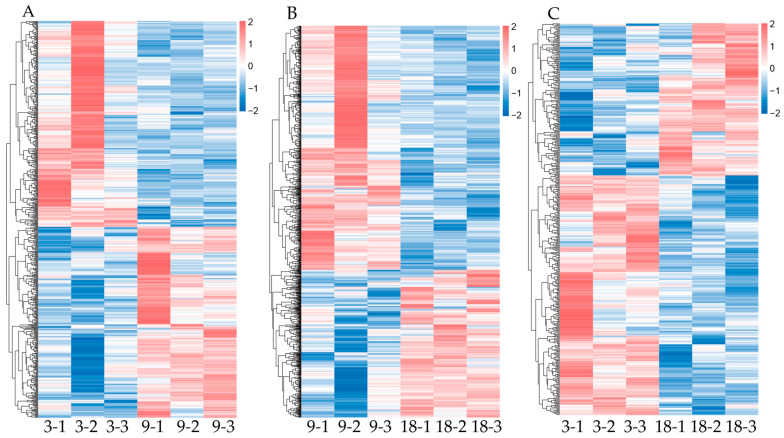
Cluster analysis of DEGs. (**A**) Heatmap of DEGs at the 3-month-old stage vs. the 9-month-old stage. (**B**) Heatmap of DEGs at the 9-month-old stage vs. the 18-month-old stage. (**C**) Heatmap of DEGs at the 3-month-old stage vs. the 18-month-old stage. The color depth directly reflects the expression level of the gene in the corresponding sample which is quantified by log10 (FPKM + 0.000001).

A total of 1395 DEGs were identified in the analysis of nine samples spanning three age groups (Figure 1). In the 3 months vs. 9 months comparison, the top five significantly downregulated genes were *RORA*, *BST-2B*, *ACTC1*, *LOC105605916*, and *ANKRD1*, while the top five significantly upregulated genes were *PARVB*, *CCL22*, *C1QTNF9*, *TIMP4*, and *IL7R*. In the 9 months vs. 18 months comparison, the top five significantly downregulated genes were *ACTC1*, *PI15*, *PTGES*, *COL14A1*, and *KIAAI755*, while the top five significantly upregulated genes were *RORA*, *FOSB*, *NR4A3*, *GSTA1*, and *AJM1*. In the 3 months vs. 18 months comparison, the top five significantly downregulated genes were *ACTC1*, *BST-2B*, *IFI6*, *CHI3L1*, and *LOC101102155*, while the top five significantly upregulated genes were *GREB1*, *TFCP2L1*, *LOC1141-8841*, *LOC101112843*, and *EFHB* (Table 6). An analysis of the identified DEGs is shown through the volcano plots and clusters in Figure 2 and Figure 3.

### 3.6. GO and KEGG Pathway Analyses of DEGs

**Figure 4:**
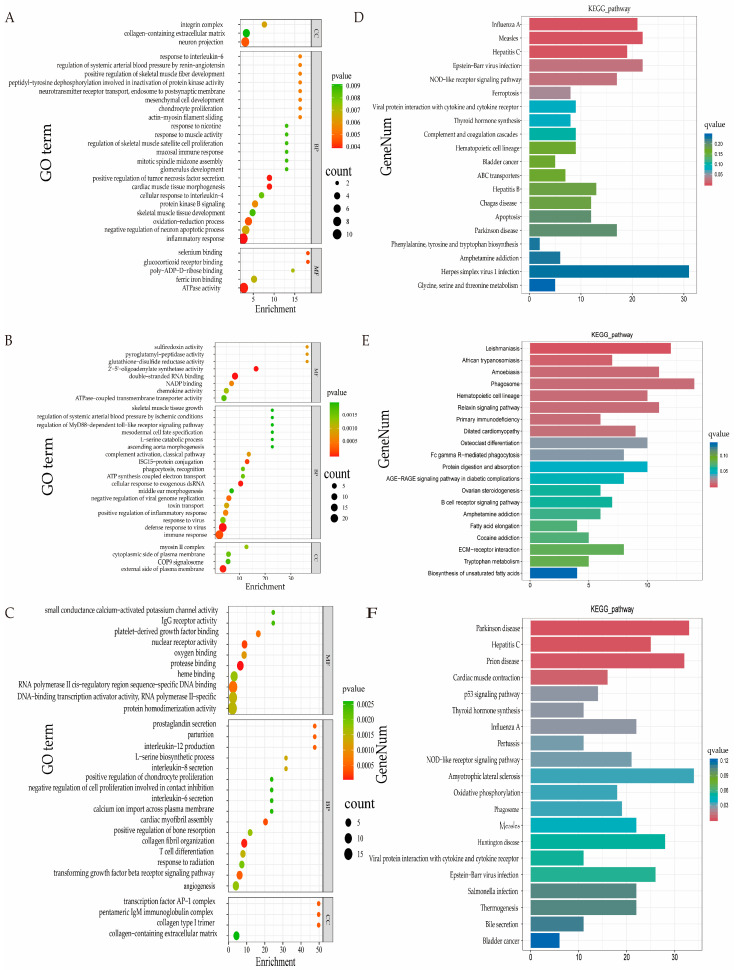
GO and KEGG enrichment analysis of DEGs at different ages (3, 9, and 18 months). (**A**) Dot plot of GO enrichment analysis of DEGs at the 3-month-old stage vs. the 9-month-old stage. (**B**) Dot plot of GO enrichment analysis of DEGs at the 9-month-old stage vs. the 18-month-old stage. (**C**) Dot plot of GO enrichment analysis of DEGs at the 3-month-old stage vs. the 18-month-old stage. (**D**) Bar plot of KEGG enrichment analysis of DEGs at the 3-month-old stage vs. the 9-month-old stage. (**E**) Bar plot of KEGG enrichment analysis of DEGs at the 9-month-old stage vs. the 18-month-old stage. (**F**) Bar plot of KEGG enrichment analysis of DEGs at the 3-month-old stage vs. the 18-month-old stage.

Functional enrichment analysis of DEGs using GO revealed that 329, 434, and 420 functional terms were significantly enriched in the following groups: 3 months vs. 9 months, 9 months vs. 18 months, and 3 months vs. 18 months, respectively (*p* < 0.05). Moreover, the terms 52, 74, and 65 were highly significantly enriched for these groups (*p* < 0.01). The top 30 GO terms with the highest levels of significance in each group are shown in Figure 4, among which functional terms such as “skeletal muscle tissue growth” and “mesodermal cell fate specification” were significantly enriched across all three age groups. Among the highly significantly enriched GO terms, 17 pathways related to growth and development included the positive regulation of skeletal muscle fiber development, skeletal muscle tissue growth, and the TGF-β receptor signaling pathway. Fifteen pathways related to meat quality traits included trivalent iron binding, redox processes, and inflammatory responses. Genes such as *SIX2*, *ACTC1*, *ANKRD1*, *GPC3*, and *MYOG* were found to be involved in multiple functional terms related to tissue and organ growth and development, while genes such as *TFRC*, *ACOT7*, *COL1A2*, and *TLR2* participated in multiple functional terms related to meat quality indicators and fatty acid metabolism. These genes showed significant expression differences across different age groups (Table 7) and may play roles in different growth stages of Huanghuai sheep.

KEGG pathway analysis of DEGs in the three age groups revealed significant enrichment in the 280, 283, and 304 pathways for the 3 months and 9 months, 9 months and 18 months, and 3 months and 18 months age groups, respectively (*p* < 0.05), with highly significant enrichment in the 14, 9, and 9 pathways (*p* < 0.01). The top 20 significant KEGG pathways were selected and are plotted as a bar chart in Figure 4. Among these, five pathways, including thyroid hormone synthesis and osteoclast differentiation, were related to growth and development. Additionally, four pathways, such as ECM–receptor interaction and fatty acid elongation, were associated with the regulation of lipogenesis, oxidative stability, and muscle structural integrity, and were significantly enriched across different age groups (Table 8).

### 3.7. qRT-PCR Validation

**Figure 5:**
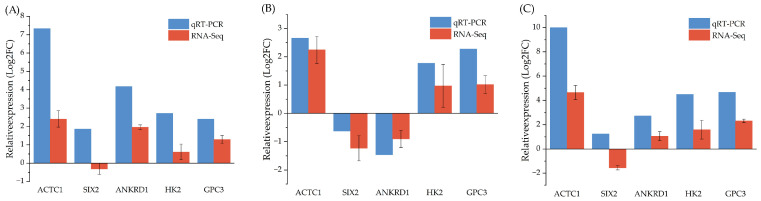
The validation results of qRT-PCR. (**A**) qPCR validation of DEGs at the 3-month-old stage vs. the 9-month-old stage. (**B**) qPCR validation of DEGs at the 9-month-old stage vs. the 18-month-old stage. (**C**) qPCR validation of DEGs at the 3-month-old stage vs. the 18-month-old stage.

To validate the reliability of the transcriptome data, five DEGs (*ACTC1*, *SIX2*, *ANKRD1*, *HK2*, and *GPC3)* were randomly selected for qRT-PCR analysis. The results showed that *ACTC1*, *ANKRD1*, *HK2*, and *GPC3* were consistent with the RNA-seq data, whereas the expression of *SIX2* was inconsistent (Figure 5).

### 3.8. Correlation Analysis Between Genes Related to Growth, Development, and Meat Quality Traits

Functional annotation was performed on the GO terms and KEGG pathway enrichment DEG results in the three age groups. The results of this enrichment analysis were used to identify the top 30 significantly enriched GO terms and the top 20 significantly enriched KEGG pathways, which were used to screen DGEs related to the growth, development, and meat quality traits in Huanghuai sheep. Subsequently, correlation analysis was conducted between these genes and the growth, development, and meat quality trait indicators. As demonstrated in Table 9, *SIX2* exhibited either a highly significant or significant correlation with chest circumference, cannon circumference, body weight, and carcass weight in Huanghuai sheep (*p* < 0.01/*p*< 0.05). Similarly, *HK2* demonstrated highly significant or significant correlations with body length, cannon circumference, and slaughter rate (*p* < 0.01/*p* < 0.05). Furthermore, a significant correlation was observed between *HSPA5* and both body length and slaughter rate (*p* < 0.05), while *COL1A2* was found to be significantly correlated with tenderness (*p* < 0.05) (Table 10). As shown in Table 11, *TLR2* exhibited significant correlations with C18:0 and C18:1n9c (*p* < 0.05). Similarly, *CHI3L1* demonstrated highly significant correlations with C18:0 and C18:1n9c (*p* < 0.01), and a significant correlation with C18:3n3 (*p* < 0.05). Furthermore, *ACOT7* was found to be significantly correlated with C18:2n6c, C20:2, and C20:4n6 (*p* < 0.05).

## 4. Discussion

This study systematically reveals the patterns of growth, carcass traits, and meat quality changes in Huanghuai sheep at three key developmental stages (3, 9, and 18 months) by integrating phenotypic data with transcriptome analysis, and preliminarily deciphered the underlying molecular mechanisms. The main findings indicate that 9 months is the economically optimal slaughter period for Huanghuai sheep. At this age, the dressing percentage reaches 51.56% (significantly higher than 47.99% at 3 months, *p* < 0.05), the marbling score is 1.83 (superior to 3 months), and the oleic acid content is 43.97% (higher than 37.00% at 3 months, *p* < 0.05). While dressing percentage is lower than 61.23% at 18 months, shear force and pH show no significant differences across groups, balancing meat quality and feeding cost. Transcriptome analysis further identifies key genes and pathways associated with these important traits.

Our data reveal age-specific expression patterns of *ACTC1* and *SIX2* correlated with growth traits (e.g., chest circumference, body weight), suggesting their potential role in the developmental regulation of muscle growth. The observed age-dependent decrease in *ACTC1* expression (*p* < 0.05), a core component of the actin family, aligns with developmental patterns documented in cattle [16] and poultry [17]. As a key constituent of myofibrils, *ACTC1* is critical for muscle fiber formation by regulating the assembly of the actin cytoskeleton [18]. Furthermore, it helps maintain the activity of Pax7+ myogenic progenitor cells, thereby promoting muscle regeneration after injury [19]. Consequently, the reduction in *ACTC1* expression may be linked to a transition from active myogenesis to hypertrophy. Concurrently, the positive correlation between *SIX2* expression and traits such as chest circumference and body weight aligns with the known regulatory functions of the *SIX* gene family (e.g., *SIX1*/*SIX4*) in fast-twitch fiber differentiation and skeletal development [20,21]. These results collectively demonstrate that the expression patterns of *ACTC1* and *SIX2* are correlated with distinct stages of muscle development. Based on this correlation, we propose that their expression patterns may be linked to the regulation of meat production performance.

Regarding meat quality, the correlation between the expression of genes such as *TLR2* and *CHI3L1* and the content of specific fatty acids (stearic and oleic acid) suggests a potential mechanistic link between innate immune signaling, extracellular matrix remodeling, and fat deposition in muscle tissue. Previous studies have established *TLR2* as a central hub linking fatty acid metabolism and immune signaling [22]. Its activity is modulated by different fatty acid classes: it is activated by saturated fatty acids (e.g., palmitate) [23,24], but inhibited by unsaturated ones (e.g., oleate, DHA). Consequently, the activation status of *TLR2* is fine-tuned via the local fatty acid profile, which in turn influences meat quality traits such as oxidative stability and tenderness. *CHI3L1* (YKL-40) acts as a significant nexus integrating metabolic and inflammatory processes. Elevated serum levels of *CHI3L1* are a hallmark of metabolic syndrome, intimately linked to insulin resistance and leptin dysregulation [25,26]. It drives pathology by modulating cytokines and fatty acid metabolism [27], notably via the IL-13Rα2/MAPK/ERK pathway, to promote inflammation and fat accumulation [28]. Our data support the view that *TLR2* and *CHI3L1* may participate in a coordinated process associated with the composition and deposition of fatty acids in muscle. This regulatory interplay may contribute to the modulation of fatty acid composition and meat quality characteristics in Huanghuai sheep. Furthermore, the *ACOT7* gene was enriched in the fatty acid elongation pathway and associated with linoleic acid and arachidonic acid content. This is consistent with its known function in hydrolyzing acyl-CoA to coordinate fatty acid biosynthesis and oxidation [29], which is critical for fat deposition, glucose homeostasis [30], and overall metabolic health.

The primary biological significance of this study lies in outlining the molecular regulatory network underlying the superior traits of Huanghuai sheep. We found that genes from three functional modules—muscle development (e.g., *ACTC1*, *SIX2*), energy metabolism (e.g., *HK2*), and lipid metabolism—constitute a complex interaction network rather than acting in isolation. For instance, the energy metabolism status might indirectly regulate the expression of genes involved in lipid metabolism by influencing the tissue microenvironment, collectively determining growth rate and meat quality.

These findings provide a preliminary basis for exploring molecular breeding strategies in Huanghuai sheep. The correlation between *ACTC1* and *SIX2* expression and early growth traits nominates them as candidate loci for further investigation. Similarly, the association of *TLR2*, *CHI3L1*, and *ACOT7* with fatty acid composition suggests their potential involvement in shaping meat quality attributes. Future studies estimating heritability and validating these associations in independent breeding populations are essential before any application in selection programs can be considered.

We acknowledge several limitations of this study. First, the analysis was confined to the *longissimus dorsi* muscle; differential expression patterns may vary across other muscle types, limiting the generalizability of our findings. Second, our transcriptomic approach focused solely on mRNA sequencing, overlooking the potential roles of non-coding RNAs and post-transcriptional regulation, which could be elucidated by integrated multi-omics approaches. Third, although key candidate genes were identified, they were validated only at the mRNA level, and their corresponding protein-level changes and physiological functions remain to be confirmed. Finally, the sample size, particularly for slaughter and transcriptomic analyses, was limited, and further validation in larger and more diverse populations is necessary to assess the robustness and breeding value of these candidate markers. Moreover, this study primarily revealed correlations between genes and traits; the specific causal regulatory mechanisms await functional validation.

Based on these limitations, future research should focus on the following: (1) expanding transcriptomic profiling to include multiple muscle types and integrating non-coding RNA and proteomic analyses; (2) validating the expression and function of key candidate genes (e.g., *ACTC1*, *TLR2*) at the protein level; (3) verifying the breeding value of potential molecular markers in independent and larger Huanghuai sheep populations to facilitate marker-assisted selection; and (4) utilizing cell-based or animal models to investigate the molecular mechanisms of core genes in muscle development and lipid metabolism.

## 5. Conclusions

This study compared the growth, slaughter performance, and meat quality traits of Huanghuai sheep at different ages. We found that key productive traits such as body size, body weight, and dressing percentage increased progressively with age. The 9-month-old stage emerged as the commercially optimal slaughter point, offering a compelling combination of a satisfactory dressing percentage, premium meat quality, and a desirable fatty acid composition. Through transcriptomic profiling, we have identified a suite of candidate genes—including *ACTC1*, *ANKRD1*, and *SIX2* associated with muscle development, and *COL1A2*, *TLR2*, *CHI3L1*, and *ACOT7* linked to meat quality and lipid metabolism. These findings contribute to a deeper understanding of the molecular factors underlying economically essential traits in Huanghuai sheep and highlight several candidate genes (e.g., *ACTC1*, *SIX2* for muscle growth; *TLR2* for lipid metabolism) that may serve as markers for future breeding efforts. Future research should employ CRISPR-Cas9-mediated functional validation of *ACTC1*/*SIX2* in muscle development, verify *TLR2*’s regulatory role in fatty acid metabolism, and integrate protein assays with metabolomics in larger cohorts to confirm these genes’ translational potential and elucidate causal mechanisms.

## Figures and Tables

**Table 1:** Real-time fluorescent quantitative primer sequence.

Genes	Primer Sequences	Product Length	Genbank Accession No.
*ACTC1*	F: ATTATTGCTCCCCCTGAGCG R: TGAGAGATGAGGGAGGGTGG	231 bp	NM_001034585.2
*ANKRD1*	F: AGCCCAGATCGAATTCCGTG R: GCGGTGCTGAGCAACTTATC	138 bp	NM_001252178.1
*SIX2*	F: ACACAGGTCAGCAACTGGTTCAAG R: GAGTTCTCGCTGTTCTCCCTTTCC	83 bp	NM_001205678.2
*GPC3*	F: CAAGAACTCGTGGAGAGATAC R: CACTTTCATCATTCCATCGC	273 bp	XM_015105024.4
*HK2*	F: TGACGGCACAGAGAAAGGAGACT R: GCACACGCACCAGCAGGA	77 bp	XM_015094476.2
*GAPDH*	F: GCAAGTTCCACGGCACAG R: GGTTCACGCCCATCACAA	249 bp	AJ431207

**Table 2:** Determination of growth and development traits of Huanghuai sheep at different ages.

Sample	Number	Body Height/cm	Body Length/cm	Chest Circumference/cm	Cannon Circumference/cm	Body Weight/kg
3	20	58.90 ± 1.43 ^Cc^	63.06 ± 1.34 ^Cc^	68.18 ± 3.57 ^Cc^	6.98 ± 0.06 ^Cc^	26.01 ± 3.77 ^Cc^
9	20	69.24 ± 1.97 ^Bb^	80.58 ± 2.03 ^Bb^	94.29 ± 2.03 ^Bb^	9.24 ± 1.46 ^Bb^	53.30 ± 2.57 ^Bb^
18	20	78.26 ± 1.42 ^Aa^	90.56 ± 4.76 ^Aa^	111.34 ± 5.44 ^Aa^	10.32 ± 0.21 ^Aa^	98.25 ± 13.84 ^Aa^
*p* value		<0.0001	<0.0001	<0.0001	<0.0001	<0.0001

Note: For the same column of data, different uppercase/lowercase letters indicate highly significant (*p* < 0.01)/significant differences (*p* < 0.05), respectively.

**Table 3:** Determination of meat quality of Huanghuai sheep at different ages.

Sample	Number	Carcass Weight/kg	Dressing Percentage/%	Marbling	Ph	Tenderness/N	GR Value/Mm	Intramuscular Fat/(G/100 g)
3	3	23.67 ± 3.85 ^Aa^	47.99 ± 1.89 ^Aa^	1.33 ± 0.28 ^Aa^	6.66 ± 0.20 ^Ab^	25.86 ± 1.42 ^Aa^	9.50 ± 2.65 ^Aa^	1.27 ± 0.65 ^Aa^
9	6	44.63 ± 3.94 ^Ab^	51.56 ± 3.86 ^Aa^	1.83 ± 0.25 ^Bb^	6.36 ± 0.11 ^Aa^	27.46 ± 6.58 ^Aa^	18.11 ± 4.25 ^Ab^	1.55 ± 0.64 ^Aa^
18	3	125.40 ± 20.5 ^Bc^	61.23 ± 2.15 ^Bb^	2.83 ± 0.14 ^Cc^	6.34 ± 0.11 ^Aa^	28.59 ± 1.33 ^Aa^	40.00 ± 8.66 ^Bc^	2.33 ± 1.42 ^Aa^
*p* value		<0.0001	<0.0014	<0.0001	0.0227	0.8013	0.0002	0.3536

Note: For the same column of data, different uppercase/lowercase letters indicate highly significant (*p* < 0.01)/significant differences (*p* < 0.05), respectively.

**Table 4:** Fatty acid content in Huanghuai sheep at different ages.

Sample	Number	C14:0	C16:0	C16:1	C18:0	C18:1n9c	C18:2n6c	C18:3n3	C20:1	C20:2	C20:4n6	C24:0	C23:0
3	3	2.49 ± 0.53 ^Aa^	21.77 ± 1.86 ^Aa^	1.65 ± 0.41 ^Aa^	17.63 ± 2.32 ^Aa^	37.00 ± 3.07 ^Ab^	7.58 ± 1.20 ^Aa^	0.30 ± 0.03 ^Aa^	0.32 ± 0.14 ^Aa^	0.82 ± 0.15 ^Aa^	4.36 ± 3.13 ^Aa^	0.81 ± 0.24 ^Aa^	0.41 ± 0.07 ^Aa^
9	6	1.84 ± 0.31 ^Aa^	21.53 ± 0.33 ^Aa^	2.26 ± 0.44 ^Aa^	15.50 ± 1.42 ^Aa^	43.97 ± 1.94 ^Aa^	7.43 ± 0.92 ^Aa^	0.32 ± 0.05 ^Aa^	0.16 ± 0.02 ^Aa^	0.52 ± 0.17 ^Ba^	3.26 ± 0.73 ^Aa^	0.43 ± 0.09 ^Aa^	0.35 ± 0.03 ^Aa^
18	3	1.49 ± 0.16 ^Aa^	24.07 ± 1.15 ^Aa^	2.21 ± 0.40 ^Aa^	14.27 ± 0.66 ^Aa^	46.03 ± 2.58 ^Aa^	6.44 ± 1.16 ^Aa^	0.33 ± 0.02 ^Aa^	0.00 ± 0.00	0.25 ± 0.06 ^Ba^	1.86 ± 0.72 ^Aa^	0.25 ± 0.06 ^Ab^	0.19 ± 0.04 ^Bb^
*p* value		0.0867	0.1702	0.3399	0.1887	0.0289	0.5601	0.6710	0.2927	0.0154	0.4671	0.0279	0.0138

Note: For the same column of data, different uppercase/lowercase letters indicate highly significant (*p* < 0.01)/significant differences (*p* < 0.05), respectively.

**Table 5:** Comparison of sequenced transcriptome sequences with reference sequences.

Sample		Total Raw Reads	Clean Reads	Clean Reads Q30 Data/%	Clean Reads NO.	Mapped Reads Rate (%)	Uniquely Mapped Ratio (%)
3	1	24,786,184	24,336,459	94.94	48,672,918	97.36	40,071,244 (91.95%)
	2	26,379,058	25,804,452	94.89	51,608,904	97.51	40,067,318 (92.37%)
	3	22,891,194	22,516,219	94.62	45,032,438	97.25	47,969,006 (90.21%)
9	1	24,255,211	23,854,695	94.25	47,709,390	97.36	44,950,518 (92.35%)
	2	23,786,569	23,435,178	94.77	46,870,356	97.40	48,227,451 (93.45%)
	3	21,628,785	21,311,159	94.79	42,622,318	97.30	41,620,548 (92.42%)
18	1	22,170,943	21,788,670	94.69	43,477,340	97.25	44,053,786 (92.34%)
	2	22,103,817	21,689,418	94.89	43,378,836	97.34	43,223,028 (92.22%)
	3	26,963,811	26,588,666	95.20	53,177,332	97.90	39,490,629 (92.65%)
average		23,885,064	23,480,546	94.78	46,949,981	97.41	43,297,059(92.22%)

Note: Q30(%): The percentage of bases whose base recognition accuracy reaches or exceeds 99.9%.

**Table 6:** The top 5 DEGs between different age groups.

Group	Expression	Genes	*p*-Value	Log_2_FoldChange
3 vs. 9	Down-regulated gene	*RORA*	3.964 × 10^−19^	−4.420
		*BST-2B*	2.297 × 10^−8^	−3.222
		*ACTC1*	1.754 × 10^−7^	−4.908
		*LOC105605916*	2.095 × 10^−7^	−4.016
		*ANKRD1*	2.214 × 10^−7^	−2.561
	Up-regulated genes	*PARVB*	2.662 × 10^−7^	1.012
		*CCL22*	1.193 × 10^−6^	3.082
		*C1QYNF9*	1.924 × 10^−5^	1.618
		*TIMP4*	2.295 × 10^−5^	1.299
		*IL7R*	2.321 × 10^−5^	1.356
9 vs. 18	Down-regulated genes	*ACTC1*	9.424 × 10^−16^	−2.102
		*PI15*	1.502 × 10^−13^	−2.289
		*PTGES*	1.718 × 10^−13^	−2.934
		*COL14A1*	4.848 × 10^−10^	−2.203
		*KIAA1755*	1.061 × 10^−7^	−1.621
	Up-regulated genes	*RORA*	1.587 × 10^−25^	4.371
		*FOSB*	2.543 × 10^−16^	3.000
		*NR4A3*	4.466 × 10^−12^	3.236
		*GSTA1*	6.901 × 10^−11^	1.826
		*AJM1*	6.583 × 10^−9^	1.726
3 vs. 18	Down-regulated genes	*ACTC1*	3.060 × 10^−15^	−6.997
		*BST-2B*	1.618 × 10^−11^	−4.117
		*IFI6*	7.357 × 10^−10^	−4.561
		*CHI3L1*	2.463 × 10^−9^	−1.704
		*LOC101102155*	2.874 × 10^−9^	−2.236
	Up-regulated genes	*GREB1*	9.820 × 10^−12^	4.375
		*TFCP2L1*	1.049 × 10^−10^	5.748
		*LOC114108841*	7.586 × 10^−10^	3.773
		*LOC101112843*	1.448 × 10^−7^	2.636
		*EFHB*	2.281 × 10^−7^	3.290

**Table 7:** GO enrichment analysis.

Category		Term	GO.ID	DEGs	*p*-Value	Up Gene	Down Gene
3 vs. 9	BP	cardiac muscle tissue morphogenesis	GO:0042474	3	0.001901		*ACTC1*, *ANKRD1*, *LOC101108995*
	BP	inflammatory response	GO:0006954	11	0.003889	*ACKR2*, *CCL22*, *CCR4*, *NLRP1*, *TSPAN2*	*CHI3L1*, *LOC101110922*, *LOC101111911*, *LOC101120093*, *LOC101123672*,
	BP	positive regulation of tumor necrosis factor secretion	GO:1904469	3	0.003934	*FZD5*	*DDX58*, *IFIH1*
	BP	positive regulation of skeletal muscle fiber development	GO:0048743	2	0.003935		*MYOG*, *BCL2*
	BP	oxidation-reduction process	GO:0055114	6	0.004503	*C5H5orf63*, *COQ10A*, *FRRS1*, *LOC101106806*	*DUOX1*, *PIR*
	BP	actin-myosin filament sliding	GO:0033275	2	0.005406	*MYBPC1*	*ACTC1*
	MF	ferric iron binding	GO:0008199	4	0.006857		*FTL*, *LOC101117015*, *LOC114109119*, *LOC121820289*
	BP	regulation of skeletal muscle satellite cell proliferation	GO:0014842	2	0.008828		*MYOG*, *LOC114109370*
9 vs. 18	BP	transforming growth factor beta receptor signaling pathway	GO:0007179	6	0.0042438	*JUN*, *WFIKKN2*	*COL1A2*, *COL3A1*, *HPGD*, *SMAD6*
	CC	collagen-containing extracellular matrix	GO:0062023	6	0.002598	*LOC101119218*, *SERPINE1*	*COL14A1*, *GPC3*, *PXDN*, *POSTN*
3 vs. 18	BP	mesodermal cell fate specification	GO:0035910	2	0.001917	*EYA1*, *SIX2*	
	BP	skeletal muscle tissue growth	GO:0062023	2	0.001917		*CHRNA1*, *CHRND*
	CC	collagen-containing extracellular matrix	GO:0042474	6	0.009131	*HMCN2*, *PLSCR1*, *SERPINE1*	*ADAMTS2*, *PLSCR1*, *SERPINE1*

**Table 8:** KEGG metabolic pathway significantly enriched by DEGs.

Month-Old Group	Pathway ID	KO ID	DEGs	*p*-Value	Up Gene	Down Gene
3 vs. 9	Ferroptosis	ko04216	8	0.000913	*STEAP3*, *TFRC*	*ACSL4*, *FTL*, *LOC101117015*, *LOC114109119*, *LOC121820289*, *SLC3A2*
	Thyroid hormone synthesis	ko04710	8	0.002335		*DUOX1*, *GPX3*, *GSR*, *HSPA5*, *LOC101117153*, *LOC121816035*, *PDIA4*, *TTF2*
	Hematopoietic cell lineage	ko04640	8	0.007082	*CD3G*, *FLT3*, *IL7R*, *ITGA1*, *LOC101106528*, *LOC101110634*, *TFRC*	*LOC105614340*
9 vs. 18	Hematopoietic cell lineage	ko04640	8	0.000207	*LOC101113211*	*FCGR1A*, *IL3RA*, *KIT*, *LOC101102230*, *LOC101109746*, *LOC101111409*, *TFRC*
	Osteoclast differentiation	ko04380	10	0.000995	*FOS*, *FOSB*, *JUN*, *JUNB*	*BTK*, *FCGR1A*, *FCGR3A*, *LCK*, *LOC105602100*, *SOCS3*
	Protein digestion and absorption	ko04974	9	0.001992	*COL23A1*, *LOC114112700*	*COL14A1*, *COL1A1*, *COL1A2*, *COL3A1*, *COL6A6*, *EMILIN3*, *SLC7A8*
	Fatty acid elongation	ko00062	4	0.004381	*LOC101104407*, *THEM4*	*ACOT7*, *HACD1*
	ECM-receptor interaction	ko04512	7	0.005454	*LOC114112700*	*COL1A1*, *COL1A2*, *COL6A6*, *FRAS1*, *FREM1*, *LOC101102230*
3 vs. 18	Cardiac muscle contraction	ko04260	16	7.58 × 10^−5^	*ATP2A2*, *KEF53_p01*, *KEF53_p07*, *KEF53_p10*, *KEF53_p11*, *LOC101121285*, *LOC101121420*, *LOC121817553*, *TPM2*, *TPM3*	*ACTC1*, *ATP1B3*, *CASQ2*, *LOC114115573*, *LOC114117869*, *TNNI3*
	Thyroid hormone synthesis	ko04918	11	0.000619	*ATF4*, *CD3G*	*ATP1B3*, *DUOX1*, *GSR*, *HSP90B1*, *HSPA5*, *LOC101117153*, *LOC114117869*, *LOC121816035*, *PDIA4*
	Oxidative phosphorylation	ko00190	18	0.001483	*KEF53_p01~13(except 06)*, *LOC101121285*, *LOC101121420*, *LOC121817553*, *NDUFA4*	*ATP6V0A1*, *LOC114115573*

**Table 9:** Correlation analysis between genes and growth and development traits.

Genes	Body Height	Body Length	Chest Circumference	Canno Round	Body Weight	Carcass Weight	Dressing Percentage
*ACTC1*	−0.840	−0.967	−0.780	−0.793	−0.787	−0.928	−0.948
*ANKRD1*	−0.654	−0.856	−0.573	−0.591	−0.561	−0.787	−0.913
*SIX2*	−0.993	−0.908	−1.000 **	−0.999 *	−1.000 **	−1.000 **	−0.952
*GPC3*	−0.932	−0.994	−0.885	−0.986	−0.881	−0.856	−0.992
*HK2*	−0.978	−0.999 *	−0.947	−1.000 **	−0.945	−0.927	−0.999 *
*HSPA5*	−0.943	−0.997 *	−0.904	−0.992	−0.883	−0.877	−1.000 *

Note: * represents 0.01 < *p* ≤ 0.05, and ** represents *p* ≤ 0.01.

**Table 10:** Correlation analysis between genes and meat quality core indicators.

Genes	Marbling	pH	Tenderness	GR Value/mm
*COL1A2*	0.855	−0.994	0.998 *	0.787
*TFRC*	−0.946	0.55	−0.692	−0.977

Note: * represents 0.01 < *p* ≤ 0.05.

**Table 11:** Correlation analysis between genes and core lipid composition indicators in meat quality.

Genes	IMF	C14:0	C16:0	C16:1	C18:0	C18:1n9c	C18:2n6c	C18:3n3	C20:1	C20:2	C20:4n6	C24:0	C23:0
*TLR2*	−0.846	0.982	−0.827	−0.979	0.997 *	−0.998 *	0.936	−0.991	0.828	0.944	0.909	0.986	0.809
*CHI3L1*	−0.879	0.992	−0.861	−0.963	1.000 **	−1.000 **	0.957	−0.998 *	0.863	0.964	0.934	0.994	0.846
*ACOT7*	−0.985	0.98	−0.978	−0.828	0.952	−0.946	1.000*	−0.968	0.978	0.999 *	0.999 *	0.976	0.971

Note: * represents 0.01 < *p* ≤ 0.05, and ** represents *p* ≤ 0.01.

## Data Availability

The data presented in this study are available on request from the corresponding author.

## References

[1] Huang C., Blecker C., Chen L., Xiang C., Zheng X., Wang Z., Zhang D. (2023). Integrating identification and targeted proteomics to discover the potential indicators of postmortem lamb meat quality. Meat Sci..

[2] Han Y., Akhtar M.F., Chen W., Liu X., Zhao M., Shi L., Khan M.Z., Wang C. (2025). Potential candidate genes influencing meat production phenotypic traits in sheep: A review. Front. Vet. Sci..

[3] Khan R., Li A., Raza S.H.A. (2022). Editorial: Genetic Regulation of Meat Quality Traits in Livestock Species. Front. Genet..

[4] Huang Y., Zhao M., Zhang X., Wei H., Liu L., Zhang Z., Cheng X., Wang G., Ren C. (2023). Indoor feeding combined with restricted grazing time improves body health, slaughter performance, and meat quality in Huang-huai sheep. Anim. Biosci..

[5] Quan K., Li J., Han H., Wei H., Zhao J., Si H.A., Zhang X., Zhang D. (2020). Review of Huang-huai sheep, a new multiparous mutton sheep breed first identified in China. Trop. Anim. Health Prod..

[6] Lou M., Zhang S., Yang W., Li S., Cao H., Zhang Z., Ling Y. (2025). Transcriptome analysis revealed the mechanism of skeletal muscle growth and development in different hybrid sheep. Anim. Biosci..

[7] Yun Y., Wu R., He X., Qin X., Chen L., Sha L., Yun X., Nishiumi T., Borjigin G. (2023). Integrated Transcriptome Analysis of miRNAs and mRNAs in the Skeletal Muscle of Wuranke Sheep. Genes.

[8] Mohammadinejad F., Mohammadabadi M., Roudbari Z., Sadkowski T. (2022). Identification of Key Genes and Biological Pathways Associated with Skeletal Muscle Maturation and Hypertrophy in *Bos taurus*, *Ovis aries*, and *Sus scrofa*. Animals.

[9] Xiao C., Wei T., Liu L.X., Liu J.Q., Wang C.X., Yuan Z.Y., Ma H.H., Jin H.G., Zhang L.C., Cao Y. (2021). Whole-Transcriptome Analysis of Preadipocyte and Adipocyte and Construction of Regulatory Networks to Investigate Lipid Metabolism in Sheep. Front. Genet..

[10] Bao G., Li S., Zhao F., Wang J., Liu X., Hu J., Shi B., Wen Y., Zhao L., Luo Y. (2022). Comprehensive Transcriptome Analysis Reveals the Role of lncRNA in Fatty Acid Metabolism in the Longissimus Thoracis Muscle of Tibetan Sheep at Different Ages. Front. Nutr..

[11] Peng H., Hu M., Liu Z., Lai W., Shi L., Zhao Z., Ma H., Li Y., Yan S. (2022). Transcriptome Analysis of the Liver and Muscle Tissues of Dorper and Small-Tailed Han Sheep. Front. Genet..

[12] (2022). Meat Sheep Production Performance.

[13] (2022). Livestock and Poultry Meat Quality Testing—Determination of Moisture, Protein and Fat—Near-Infrared Spectroscopy Method.

[14] Ghosh S., Chan C.K. (2016). Analysis of RNA-Seq Data Using TopHat and Cufflinks. Plant Bioinformatics: Methods and Protocols.

[15] Song J., Cho J., Park J., Hwang J.H. (2022). Identification and validation of stable reference genes for quantitative real time PCR in different minipig tissues at developmental stages. BMC Genom..

[16] Li A., Su X., Tian Y., Song G., Zan L., Wang H. (2021). Effect of Actin Alpha Cardiac Muscle 1 on the Proliferation and Differentiation of Bovine Myoblasts and Preadipocytes. Animals.

[17] Gu S., Huang Q., Jie Y., Sun C., Wen C., Yang N. (2024). Transcriptomic and epigenomic landscapes of muscle growth during the postnatal period of broilers. J. Anim. Sci. Biotechnol..

[18] Zhao L., Li F., Zhang X., Tian H., Ma Z., Yang X., Zhang Q., Pu M., Cao P., Zhang D. (2025). RNA-Seq and WGCNA Identify Key Regulatory Modules and Genes Associated with Water-Holding Capacity and Tenderness in Sheep. Animals.

[19] Chen J., Neil J.A., Tan J.P., Rudraraju R., Mohenska M., Sun Y.B.Y., Walters E., Bediaga N.G., Sun G., Zhou Y. (2023). Author Correction: A placental model of SARS-CoV-2 infection reveals ACE2-dependent susceptibility and differentiation impairment in syncytiotrophoblasts. Nat. Cell Biol..

[20] Cai C., Wan P., Wang H., Cai X., Wang J., Chai Z., Wang J., Wang H., Zhang M., Yang N. (2023). Transcriptional and open chromatin analysis of bovine skeletal muscle development by single-cell sequencing. Cell Prolif..

[21] Maire P., Dos Santos M., Madani R., Sakakibara I., Viaut C., Wurmser M. (2020). Myogenesis control by SIX transcriptional complexes. Semin. Cell Dev. Biol..

[22] Sam Q.H., Ling H., Yew W.S., Tan Z., Ravikumar S., Chang M.W., Chai L.Y.A. (2021). The Divergent Immunomodulatory Effects of Short Chain Fatty Acids and Medium Chain Fatty Acids. Int. J. Mol. Sci..

[23] Hwang D.H., Kim J.A., Lee J.Y. (2016). Mechanisms for the activation of Toll-like receptor 2/4 by saturated fatty acids and inhibition by docosahexaenoic acid. Eur. J. Pharmacol..

[24] Howe A.M., Burke S., O’Reilly M.E., McGillicuddy F.C., Costello D.A. (2022). Palmitic Acid and Oleic Acid Differently Modulate TLR2-Mediated Inflammatory Responses in Microglia and Macrophages. Mol. Neurobiol..

[25] El-Mesallamy H.O., Mostafa A.M., Amin A.I., El Demerdash E. (2011). The interplay of YKL-40 and leptin in type 2 diabetic obese patients. Diabetes Res. Clin. Pract..

[26] Kyrgios I., Galli-Tsinopoulou A., Stylianou C., Papakonstantinou E., Arvanitidou M., Haidich A.B. (2012). Elevated circulating levels of the serum acute-phase protein YKL-40 (chitinase 3-like protein 1) are a marker of obesity and insulin resistance in prepubertal children. Metabolism.

[27] Yu J.E., Yeo I.J., Han S.B., Yun J., Kim B., Yong Y.J., Lim Y.S., Kim T.H., Son D.J., Hong J.T. (2024). Significance of chitinase-3-like protein 1 in the pathogenesis of inflammatory diseases and cancer. Exp. Mol. Med..

[28] Blazevic N., Rogic D., Pelajic S., Miler M., Glavcic G., Ratkajec V., Vrkljan N., Bakula D., Hrabar D., Pavic T. (2024). YKL-40 as a biomarker in various inflammatory diseases: A review. Biochem. Medica.

[29] Liu T., Feng H., Yousuf S., Xie L., Miao X. (2022). Differential regulation of mRNAs and lncRNAs related to lipid metabolism in Duolang and Small Tail Han sheep. Sci. Rep..

[30] Zhang X.Y., Yuan Z.H., Li F.D., Yue X.P. (2022). Integrating transcriptome and metabolome to identify key genes regulating important muscular flavour precursors in sheep. Animal.

